# Travel-associated international spread of Oropouche virus beyond the Amazon

**DOI:** 10.1093/jtm/taaf018

**Published:** 2025-03-02

**Authors:** Felipe Campos de Melo Iani, Felicidade Mota Pereira, Elaine Cristina de Oliveira, Janete Taynã Nascimento Rodrigues, Mariza Hoffmann Machado, Vagner Fonseca, Talita Emile Ribeiro Adelino, Natália Rocha Guimarães, Luiz Marcelo Ribeiro Tomé, Marcela Kelly Astete Gómez, Vanessa Brandão Nardy, Adriana Aparecida Ribeiro, Alexander Rosewell, Álvaro Gil A Ferreira, Arabela Leal e Silva de Mello, Brenda Machado Moura Fernandes, Carlos Frederico Campelo de Albuquerque, Dejanira dos Santos Pereira, Eline Carvalho Pimentel, Fábio Guilherme Mesquita Lima, Fernanda Viana Moreira Silva, Glauco de Carvalho Pereira, Houriiyah Tegally, Júlia Deffune Profeta Cidin Almeida, Keldenn Melo Farias Moreno, Klaucia Rodrigues Vasconcelos, Leandro Cavalcante Santos, Lívia Cristina Machado Silva, Livia C V Frutuoso, Ludmila Oliveira Lamounier, Mariana Araújo Costa, Marília Santini de Oliveira, Marlei Pickler Dediasi dos Anjos, Massimo Ciccozzi, Maurício Teixeira Lima, Maira Alves Pereira, Marília Lima Cruz Rocha, Paulo Eduardo de Souza da Silva, Peter M Rabinowitz, Priscila Souza de Almeida, Richard Lessells, Ricardo T Gazzinelli, Rivaldo Venâncio da Cunha, Sabrina Gonçalves, Sara Cândida Ferreira dos Santos, Senele Ana de Alcântara Belettini, Silvia Helena Sousa Pietra Pedroso, Sofia Isabel Rótulo Araújo, Stephanni Figueiredo da Silva, Julio Croda, Ethel Maciel, Wes Van Voorhis, Darren P Martin, Edward C Holmes, Tulio de Oliveira, José Lourenço, Luiz Carlos Junior Alcantara, Marta Giovanetti

**Affiliations:** Central Public Health Laboratory of the State of Minas Gerais, Ezequiel Dias Foundation, Belo Horizonte, Minas Gerais, 30.510-010, Brazil; Central Public Health Laboratory of the State of Bahia, Salvador, Bahia, 41745-900, Brazil; Central Public Health Laboratory of the State of Mato Grosso, Campo Grande, Mato Grosso, 78020-500, Brazil; Central Public Health Laboratory of the State of Acre, Rio Branco, Acre, 69918-504, Brazil; Central Public Health Laboratory of the State of Santa Catarina, Florianopolis, 88010-001, Brazil; Department of Exact and Earth Sciences, University of the State of Bahia, Salvador, Salvador, 45083-900, Brazil; Centre for Epidemic Response and Innovation (CERI), School of Data Science and Computational Thinking, Stellenbosh, 7602, Stellenbosch; René Rachou Institute, Oswaldo Cruz Foundation, Belo Horizonte, 30190002, Brazil; Central Public Health Laboratory of the State of Minas Gerais, Ezequiel Dias Foundation, Belo Horizonte, Minas Gerais, 30.510-010, Brazil; René Rachou Institute, Oswaldo Cruz Foundation, Belo Horizonte, 30190002, Brazil; Central Public Health Laboratory of the State of Minas Gerais, Ezequiel Dias Foundation, Belo Horizonte, Minas Gerais, 30.510-010, Brazil; René Rachou Institute, Oswaldo Cruz Foundation, Belo Horizonte, 30190002, Brazil; Central Public Health Laboratory of the State of Minas Gerais, Ezequiel Dias Foundation, Belo Horizonte, Minas Gerais, 30.510-010, Brazil; René Rachou Institute, Oswaldo Cruz Foundation, Belo Horizonte, 30190002, Brazil; Central Public Health Laboratory of the State of Bahia, Salvador, Bahia, 41745-900, Brazil; Central Public Health Laboratory of the State of Bahia, Salvador, Bahia, 41745-900, Brazil; Central Public Health Laboratory of the State of Minas Gerais, Ezequiel Dias Foundation, Belo Horizonte, Minas Gerais, 30.510-010, Brazil; Organização Pan-Americana da Saúde, Organização Mundial da Saúde, Distrito Federal, Brasilia, 70800-400, Brazil; René Rachou Institute, Oswaldo Cruz Foundation, Belo Horizonte, 30190002, Brazil; Central Public Health Laboratory of the State of Bahia, Salvador, Bahia, 41745-900, Brazil; Central Public Health Laboratory of the State of Acre, Rio Branco, Acre, 69918-504, Brazil; Organização Pan-Americana da Saúde, Organização Mundial da Saúde, Distrito Federal, Brasilia, 70800-400, Brazil; Central Public Health Laboratory of the State of Mato Grosso, Campo Grande, Mato Grosso, 78020-500, Brazil; Central Public Health Laboratory of the State of Bahia, Salvador, Bahia, 41745-900, Brazil; Central Public Health Laboratory of the State of Acre, Rio Branco, Acre, 69918-504, Brazil; Central Public Health Laboratory of the State of Minas Gerais, Ezequiel Dias Foundation, Belo Horizonte, Minas Gerais, 30.510-010, Brazil; Central Public Health Laboratory of the State of Minas Gerais, Ezequiel Dias Foundation, Belo Horizonte, Minas Gerais, 30.510-010, Brazil; Centre for Epidemic Response and Innovation (CERI), School of Data Science and Computational Thinking, Stellenbosh, 7602, Stellenbosch; Central Public Health Laboratory of the State of Mato Grosso, Campo Grande, Mato Grosso, 78020-500, Brazil; Institute of Biological Sciences, Federal University of Minas Gerais, Belo Horizonte, 31270-901, Brazil; Central Public Health Laboratory of the State of Mato Grosso, Campo Grande, Mato Grosso, 78020-500, Brazil; Central Public Health Laboratory of the State of Acre, Rio Branco, Acre, 69918-504, Brazil; Central Public Health Laboratory of the State of Minas Gerais, Ezequiel Dias Foundation, Belo Horizonte, Minas Gerais, 30.510-010, Brazil; Coordenadora-Geral de Vigilância de Arboviroses, Brazilian Ministry of Health, Distrito Federal, Brasilia, 70800-400, Brazil; Central Public Health Laboratory of the State of Minas Gerais, Ezequiel Dias Foundation, Belo Horizonte, Minas Gerais, 30.510-010, Brazil; Central Public Health Laboratory of the State of Acre, Rio Branco, Acre, 69918-504, Brazil; Coordenadora-Geral de Laboratórios de Saúde Pública, Brazilian Ministry of Health, Distrito Federal, Brasilia, 70800-400, Brazil; Central Public Health Laboratory of the State of Santa Catarina, Florianopolis, 88010-001, Brazil; Unit of Medical Statistics and Molecular Epidemiology, University Campus Bio-Medico of Rome, Rome, 00128, Italy; Institute of Biological Sciences, Federal University of Minas Gerais, Belo Horizonte, 31270-901, Brazil; Central Public Health Laboratory of the State of Minas Gerais, Ezequiel Dias Foundation, Belo Horizonte, Minas Gerais, 30.510-010, Brazil; Central Public Health Laboratory of the State of Minas Gerais, Ezequiel Dias Foundation, Belo Horizonte, Minas Gerais, 30.510-010, Brazil; Central Public Health Laboratory of the State of Minas Gerais, Ezequiel Dias Foundation, Belo Horizonte, Minas Gerais, 30.510-010, Brazil; Environmental and Occupational Health Sciences, University of Washington, Seattle, 98195, USA; Central Public Health Laboratory of the State of Minas Gerais, Ezequiel Dias Foundation, Belo Horizonte, Minas Gerais, 30.510-010, Brazil; KwaZulu-Natal Research Innovation and Sequencing Platform (KRISP), Nelson R Mandela School of Medicine, University of KwaZulu-Natal, Durban 4001, South Africa; Fundação Oswaldo Cruz—Minas, Laboratory of Immunopatology, Belo Horizonte, MG, 30.190-009, Brazil; Fundação Oswaldo Cruz, Bio-Manguinhos, Rio de Janeiro, 21.040-361, Brazil; Central Public Health Laboratory of the State of Santa Catarina, Florianopolis, 88010-001, Brazil; Central Public Health Laboratory of the State of Minas Gerais, Ezequiel Dias Foundation, Belo Horizonte, Minas Gerais, 30.510-010, Brazil; Central Public Health Laboratory of the State of Santa Catarina, Florianopolis, 88010-001, Brazil; Central Public Health Laboratory of the State of Minas Gerais, Ezequiel Dias Foundation, Belo Horizonte, Minas Gerais, 30.510-010, Brazil; Central Public Health Laboratory of the State of Santa Catarina, Florianopolis, 88010-001, Brazil; Central Public Health Laboratory of the State of Mato Grosso, Campo Grande, Mato Grosso, 78020-500, Brazil; Faculdade de Medicina, Universidade Federal de Mato Grosso do Sul, Campo Grande, MS, 79070-900, Brazil; Fundação Oswaldo Cruz—Mato Grosso do Sul, Campo Grande, MS, 79070-900, Brazil; Secretária de Vigilância em Saúde e Ambiente (SVSA—Ministério da Saúde), Distrito Federal, Brasilia, 70800-400, Brazil; Center for Emerging and Re-emerging Infectious Diseases (CERID), University of Washington, Seattle, 98195; Computational Biology Division, Department of Integrative Biomedical Sciences, Institute of Infectious Disease and Molecular Medicine, Faculty of Health Sciences, University of Cape Town, Cape Town, 7700, South Africa; School of Medical Sciences, University of Sydney, Sydney, NSW, 2050, Australia; School for Data Science and Computational Thinking, Faculty of Science and Faculty of Medicine and Health Sciences, Stellenbosch University, Stellenbosh, 7602, South Africa; Universidade Católica Portuguesa, Católica Medical School, Católica Biomedical Research Centre, Lisbon, 1649-023, Portugal; Climate amplified diseases and epidemics (CLIMADE) Europe, Lisbon, 1649-023, Portugal; René Rachou Institute, Oswaldo Cruz Foundation, Belo Horizonte, 30190002, Brazil; Department of Sciences and Technologies for Sustainable Development and One Health, Università Campus Bio-Medico di Roma, Rome, 00128, Italy; Oswaldo Cruz Institute, Oswaldo Cruz Foundation, Minas Gerais, Belo Horizonte, 30.190-009, Brazil

**Keywords:** Orov, Brazil, genomic surveillance, Amazon basin

## Abstract

Oropouche virus (OROV), first detected in Trinidad and Tobago in 1955, was historically confined to the Brazilian Amazon Basin. However, since late 2022, an increasing number of OROV cases have been reported across various regions of Brazil as well as in urban centers in Bolivia, Ecuador, Guyana, Colombia, Cuba, Panama, and Peru. In collaboration with Central Public Health Laboratories across Brazil, we integrated epidemiological metadata with genomic analyses from recent cases, generating 133 whole-genome sequences covering the virus’s three genomic segments (L, M, and S). These include the first genomes from regions outside the Amazon and from the first recorded fatal cases. Phylogenetic analyses show that the 2024 OROV genomes form a monophyletic group with sequences from the Amazon Basin sampled since 2022, revealing a rapid north-to-south viral movement into historically non-endemic areas. We identified 21 reassortment events, though it remains unclear whether these genomic changes have facilitated viral adaptation to local ecological conditions or contributed to phenotypic traits of public health significance. Our findings demonstrate how OROV has evolved through reassortment and spread rapidly across multiple states in Brazil, leading to the largest outbreak ever recorded outside the Amazon and the first confirmed fatalities. Additionally, by analysing travel-related cases, we provide the first insights into the international spread of OROV beyond Brazil, further highlighting the role of human mobility in its dissemination. The virus’s recent rapid geographic expansion and the emergence of severe cases emphasize the urgent need for enhanced surveillance across the Americas. In the absence of significant human population changes over the past two years, factors such as viral adaptation, deforestation, and climate shifts—either individually or in combination—may have facilitated the spread of OROV beyond the Amazon Basin through both local and travel-associated transmission.

## Background

Oropouche virus (OROV; *Orthobunyavirus oropoucheense*) is an arthropod-borne virus classified within the order *Bunyavirales*, family *Peribunyaviridae* and genus *Orthobunyavirus.*^1^ Its genome is a negative-sense, single-stranded RNA ~13 200–13 400 nucleotides in length, segmented into three parts based on size: S (small, ~ 0.7 kb), M (medium, ~ 4.4 kb), and L (large, ~ 6.9 kb). These segments encode essential components such as the virus RNA-dependent RNA polymerase (RdRp), viral surface glycoproteins (Gn and Gc), and nucleocapsid protein (N).^2^ OROV was first identified in 1955 in Oropouche, a village in Trinidad and Tobago.^3^ Since then, the virus has been responsible for numerous outbreaks, mostly in the Amazon Basin, where it is found among forest animals such as non-human primates, sloths, and birds.^4^ The midge *Culicoides paraensis* is the primary vector for human transmission. Infection with OROV causes Oropouche fever, which typically presents as fever, headache, muscle and joint pain.^5^ The majority of human infections present as mild to moderate disease and resolve within a week, although rare cases can lead to complications like aseptic meningoencephalitis.^6^

Historically, OROV outbreaks were largely restricted to the Amazon region, with ~30 outbreaks reported in Latin America up until August 2024.^7^ However, new epidemiological data show a marked increase in Oropouche fever cases in Brazil, Cuba (N = 74), Bolivia (N = 356), Colombia (N = 74), Ecuador (N = 3), Guyana (N = 3), Panama (16), and Peru (N = 290), with travel-related cases reported in Italy, Spain, and Germany, all originating from Cuba, highlighting the virus is spreading outside the Amazon.^8^^,^^9^ Brazil alone reported a total number of more than 7000 cases this year (up to August 2024) compared to 836 in 2023, indicating a significant increase in transmission.^10–12^ In 2024, Brazil also reported the first three fatal cases associated with OROV infection, raising concerns among health authorities.^13^ OROV has also recently appeared in historically non-endemic Brazilian regions outside the Amazon, including in the far south and, importantly, in some urban centers on the east coast.^13^ As is the case for other arboviruses,^14^ recent changes in disease ecology, such as deforestation, urbanization, human mobility, and climate change, are possible drivers of its recent emergence.^15^ Local reservoir habitats belonging to non-human mammals and vectors can be disrupted, pushing them into closer contact with each other and with urban and peri-urban areas where humans can be infected. In addition, human mobility favours long distance viral movement. Additionally, OROV evolution may also result in changes in virulence and transmission potential.^16^ Scientific and surveillance data for OROV are currently limited, with fewer than 110 peer-reviewed publications compared to thousands for other arboviruses such as Zika or Dengue.^17^ Reports of recent increases in OROV epidemic activity underscore the urgent need for more data and research. Some of the epidemic activity over the past decade has provided valuable insights into OROV epidemiological characteristics, as well as its potential for public health impact. To help fill current knowledge gaps in the face of recent epidemic activity in the south of Brazil we, in collaboration with several Central Public Health Laboratories, generated 133 viral genome sequences including all three segments (L, M, and S). Herein, we present a genomic analysis that provides insights into OROV’s recent movement from northern to southern Brazil and its international spread into regions—both within and beyond the country—that were not historically associated with epidemic activity.

## Methods

### Ethics statement

This project was reviewed and approved by the Ethical Committee of the Federal University of Minas Gerais (CAAE: 32912820.6.1001.5149). The availability of the samples for research purposes during outbreaks of national concern is allowed by the terms of the 510/2016 Resolution of the National Ethical Committee for Research (CONEP—Comissão Nacional de Ética em Pesquisa, Ministério da Saúde) of the Brazilian Ministry of Health (BrMoH), that authorize, without the necessity of an informed consent, the use of clinical samples collected in the Brazilian Central Public Health Laboratories to accelerate knowledge building and contribute to surveillance and outbreak response. The samples processed in this study were obtained anonymously from material collected during routine arboviral diagnosis in Brazilian public health laboratories within the BrMoH network.

### Sample collection and molecular diagnostic screening

Patients with suspected OROV infection presented with acute febrile illness, including mild symptoms such as arthralgia, fever, headache and myalgia. Clinical samples were collected from local health services in five Brazilian states: Minas Gerais, Bahia, Mato Grosso, Acre, and Santa Catarina, for routine diagnostic purposes. Samples were collected between February and May 2024. Viral RNA was extracted from serum samples using an automated protocol, and samples were submitted to a multiplex arbovirus molecular screening by RT-qPCR, including the detection of OROV based on an assay by Naveca et al. (2017).^18^ All of these samples yielded positive results only for OROV.

### cDNA synthesis and whole genome sequencing

Samples were selected for sequencing based on CT value (≤36) and availability of clinical and epidemiological metadata, such as date of symptom onset, date of sample collection, sex, age, municipality of residence, and symptoms. For cDNA synthesis, the ProtoScript II First Strand cDNA Synthesis kit (NEB) was used following the manufacturer’s instructions. The cDNA generated was subjected to multiplex PCR sequencing using Q5 High Fidelity Hot-Start DNA Polymerase (NEB) and a set of specific primers designed by the Zibra Project (https://github.com/zibraproject/zika-pipeline/tree/master/schemes/OROV400/V1) for sequencing the complete genomes of OROV. Whole genome sequencing was performed using both the MiSeq (Illumina) and MinION (Oxford Nanopore Technologies) instruments. In the first case, OROV library preparation was carried out using the KAPA HyperPlus kit (Roche), following the manufacturer’s instructions. The normalized library was loaded onto a 300-cycle MiSeq Reagent Micro Kit v2 and run on the MiSeq platform (Illumina). For nanopore sequencing, DNA library preparation was performed using the ligation sequencing kit LSK109 (Oxford Nanopore Technologies) and the native barcoding kit EXP-NBD196 (Oxford Nanopore Technologies). Sequencing libraries were loaded into an R9.4 flow cell (Oxford Nanopore Technologies).

### Generation of consensus sequences

Raw files were basecalled and demultiplexing was done using Guppy v.6.0 (Oxford Nanopore Technologies). Consensus sequences were generated by a hybrid approach using the Genome Detective online tool (https://www.genomedetective.com/).^19^

### Phylogenetic analysis

Sequences of the 133 complete S, M, and L genomic segments of OROV generated in this study were combined with corresponding segments of all published full-length OROV sequences available in NCBI/GenBank up to July 2024 (S = 376 sequences, M = 231 sequences, and L = 303 sequences). The sequences from two fatal cases from the state of Bahia (Brazil) collected in March and May 2024, along with one from Mato Grosso, were also included. Sequence alignment of each segment data set was performed using MAFFT^20^ and manually curated to remove artefacts using AliView.^21^ The full genome dataset (with segments concatenated) was checked for potential recombination and reassortment using the RDP5 program (employing default settings except that segments were considered as linear sequences and window sizes of 16, 50, 40 and 101 were used for the RDP, Chimaera, Maxchi and Bootscan recombination/reassortment detection methods, respectively.^22^ Genomic regions identified by RDP5 to have been acquired by recombination and genomic segments identified by RDP5 to have been acquired by reassortment, were stripped from the full genome data sets by replacing these regions with gap characters (‘-’) in the alignment file, thereby a yielding a free full genome alignment free of recombination and reassortment. The full genome alignment and those of the individual segments were used to infer Maximum Likelihood (ML) phylogenetic trees using IQ-TREE version 2^23^ under the HKY nucleotide substitution model as inferred by the ModelFinder application. Branch support was assessed using the approximate likelihood-ratio test based on bootstrap and the Shimodaira–Hasegawa-like procedure with 1000 replicates.

Three different data subsets containing only the 2022–2024 extra-Amazon sequences of S (n = 162), M (n = 162), and L (n = 162) segments, were used to infer patterns of spatiotemporal spread from continuous spatially explicit phylogeographic reconstructions using BEAST v1.10.4.^24^ Before phylogeographic analysis, the extent of molecular clock signal in each data subset was assessed using the root-to-tip regression method available in TempEst v1.5.3^25^ following the removal of potential outliers that likely violate the clock assumption. We accepted temporal structure when the correlation coefficient was > 0.2. We modelled the phylogenetic diffusion and spread of OROV within Brazil by analysing localized transmission (between Brazilian regions) using a flexible relaxed random walk diffusion model^26^ that accommodates branch-specific variation in rates of dispersal, with a Cauchy distribution and a jitter window size of 0.01.^27^ For each sequence, the latitude and longitude coordinates of the sample were employed. MCMC analyses were set up in BEAST v1.10.4, running in duplicate for 50 million interactions and sampling every 10 000 steps in the chain. Convergence for each run was assessed in Tracer v1.7.1 (effective sample size for all relevant model parameters > 200).^28^ Maximum clade credibility trees for each run were summarized using TreeAnnotator after discarding the initial 10% as burn-in. Finally, we used the R package seraphim^29^ to extract and map spatiotemporal information embedded in the posterior trees.

To better understand the global dissemination of a specific Brazilian sublineage from 2022–2024, we expanded the data set for each segment to include genome sequences recently isolated in Peru and Italy. We then estimated time-scaled global tree topologies and performed discrete ancestral state reconstruction (of locations) using the *mugration* package extension of TreeTime under a GTR model.^30^ Using a custom Python script, we tracked the number of state changes by iterating over each phylogeny from the root to the external tips. We recorded state changes whenever an internal node transitioned from one country to another in its child node or tip(s). The timing of these transition events was documented, providing estimates for import or export events.^30^

## Results

**Figure 1:**
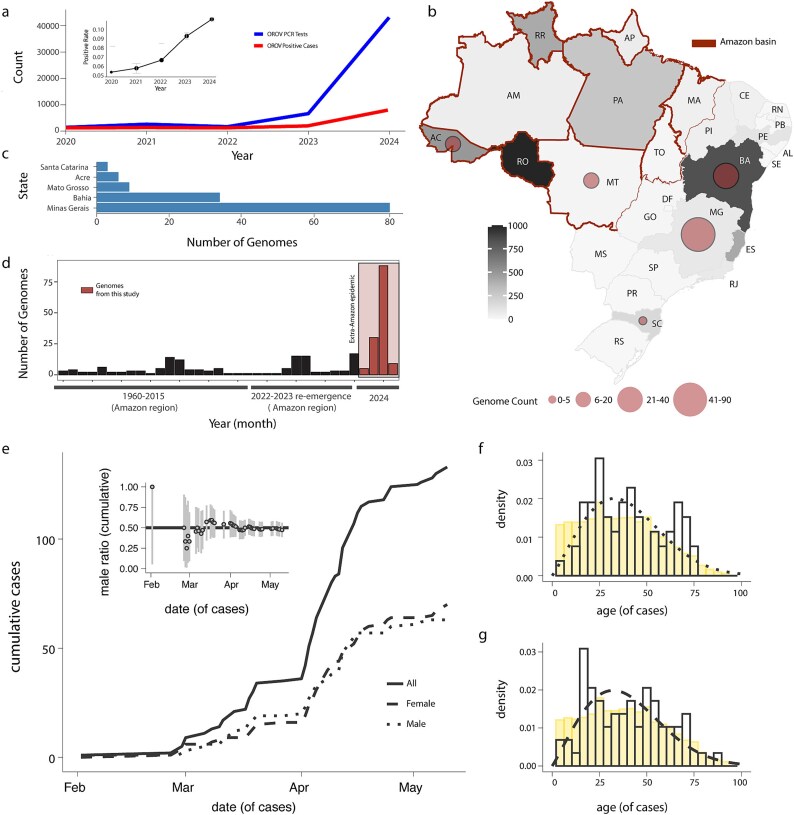
**Distribution and Epidemiological Insights of OROV Clinical Cases Detected Beyond the Amazon Basin.** (a) Weekly notified OROV PCR tests and positive cases normalized per 100 K individuals per region from 2020 to 2024. The inset panel shows the positive rate (ratio of positive cases to PCR tests) over the same period, with the y-axis scaled from 0.05 to 0.1 to highlight the variations. Error bars in the inset panel represent 95% confidence intervals calculated using Wilson's method, providing statistical support for the positive rate estimates; (b) Map of Brazil showing the number of new OROV sequences by state. The map depicts the spatial distribution of newly generated OROV genomes across Brazilian states. The size and colour of the circles represent the number of genomes generated in this study, while the shading of each state indicates the total genome count. States such as Acre (AC), Rondônia (RO), and Amazonas (AM) are shown within the Amazon Basin, while Minas Gerais (MG), Bahia (BA), and Santa Catarina (SC) represent regions beyond the Amazon Basin where sequencing efforts have expanded. This highlights the broad geographic scope of the study and the increasing efforts to monitor OROV in diverse regions of Brazil; (c) Number of new OROV genomes per state obtained in this study (Santa Catarina n = 3; Acre n = 6, Mato Grosso n = 9; Bahia n = 34; Minas Gerais n = 81); (d) Number of genomes generated in this study compared to the number of Brazilian OROV sequences available on GenBank up to 31st July, 2024. Bars are months, months with 0 sampling are not shown; (e) Cumulative OROV cases in absolute (full line) and by gender (male dotted, female dashed) of samples from 2024 for which gender metadata was available; the inner panel shows the male gender ratio (M/N) per date (grey bars are the Wilson 95% confidence interval); (f–g) Observed (non-filled bars), background (yellow bars) and theoretical (lines) age-distributions for males (f) and females (g) of samples from 2024 for which gender metadata was available (males N = 63, female N = 70). Fitted theoretical distributions were Weibull (male: shape 2.11, scale 44.39, mean 39.15; female: shape 2.07, scale 44.22, mean 39.16; determined by fitting a wide range of possible distributions using the R-package MASS). Background distributions per gender extracted from IBGE census projection for 2024.

Between late 2022 and early 2024, the Brazilian states of Acre (AC), Amazonas (AM), Rondônia (RO), and Roraima (RR), located in the western Amazon region, reported a sharp increase in OROV human cases (Fig. 1a).^11^ This rise coincided with a substantial increase in the number of real-time RT-PCR tests conducted across the country, reflecting intensified surveillance efforts (Fig. 1a).^11^ In 2020, initial screening efforts focused primarily on the northern region of Brazil, with the Roraima and Pará (PA) states accounting for the majority of the 238 tests conducted.^31^ During the same period, a small number of positive cases were detected in Amapá (AP), Amazonas, Pará, Piauí (PI), and Rondônia, indicating early OROV circulation.^31^ By 2021, the number of tests surged to 1466, with significant increases in Minas Gerais (MG) and Ceará (ce), and positive cases were reported predominantly in Amapá, Pará, and Piauí.^13^ The trend of increased testing continued into 2022, with 588 tests conducted, marking notable expansions in Midwestern and Northeastern Brazilian states.^13^ In 2023, screening efforts intensified further, with 5280 tests conducted nationwide, including significant testing numbers in Bahia (BA), Goiás (GO), Rio de Janeiro (RJ), and Tocantins (TO).^13^ Positive cases were detected in Acre, Maranhão (MA), Mato Grosso (MT), Pará, Piauí, Rio Grande do Norte *RN), Rondônia, Roraima, and Tocantins (Fig. 1a).^13^ By early 2024, screening efforts reached an unprecedented level, with 54 428 tests conducted across multiple states (Fig. 1a).^13^ The states of Espírito Santo (ES), Minas Gerais, Bahia, and Goiás reported the highest testing numbers outside the Amazon basin.^13^ Within this period, cumulative positive cases were highest in Rondônia (n = 1747), Bahia (n = 837), Espírito Santo (n = 416), and Roraima (n = 244) (Fig. 1b).^13^

Five Brazilian states were represented in the 133 newly generated genome sequences: Acre (N = 6, northwest), Bahia (N = 34, Northeast), Mato Grosso (N = 9, Midwest), Minas Gerais (N = 81, Southeast), and Santa Catarina (SC) (N = 3, South) (Fig. 1c**, a**). This sampling covered a wide spatial range outside the Amazon basin (Fig. 1a) and represents the most time-intensive sampling period to date in Brazil (Fig. 1d). The average cycle threshold (CT) value of the generated genomes was 25, ranging from 8 to 36 (Table S1). The gender ratio associated with the genome samples was biased towards females in February–March 2024 but converged to 1 into late local autumn (Fig. 1e). Cumulatively, 53% (n = 70) were female and 47% (n = 63) were male (Table S1), with genders showing a similar age profile (Fig. 1f-g). Ages ranged from 1 to 89 years, with a median age of ~ 39 years. Genome sequences were obtained from all five Brazilian macro-regions (North, South, Northeast, Southeast, and Midwest) (Fig. 1). The most frequently reported symptoms among patients were fever, myalgia, and headache, with some individuals also experiencing arthralgia (Table S1). In addition to the common symptomatic presentations, our study identified three fatal cases associated with OROV infection. Notably, these fatalities occurred in young adult patients with no reported comorbidities. In all cases, the clinical course resembled severe dengue, with shock, bleeding, and extensive coagulopathy. Point mutations were identified in three fatal cases when compared to non-fatal cases. In the M segment, amino acid changes included I to V at position 13, M to I at position 642, A to T at position 752, and R to K at position 1342. In the L segment, mutations were found at amino acid positions 857 (T to A), 1634 (K to E), and 2206 (N to D).

The sequencing procedures yielded an average coverage of 97.7% for segment S, 98.5% for segment M, and 98.32% for segment L (Table S1). Using a Generalized Additive Model, response curves were generated to estimate sequence coverage as a function of CT values. Observed and estimated sequence coverage were negatively correlated with CT values (Fig. S2a). The M segment showed the highest coverage, while the S segment had slightly lower coverage, independently of CT values. The states of Bahia and Minas Gerais (~26% and ~61% of samples, respectively) showed consistently lower sequence coverage for Bahia and higher coverage for Minas Gerais, independently of CT values and across all genome segments (Fig. S3). Prolonged intervals between symptom onset and sample collection were associated with increased CT values (Fig. S2b).

**Figure 2:**
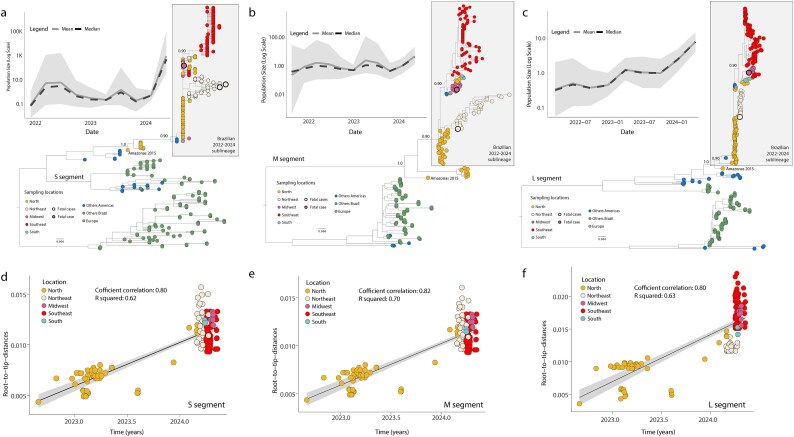
**Molecular Evolution and Demographic History of OROV Segments S, M, and L.** (a–c) Maximum likelihood phylogenetic trees of the three OROV segments: S (n = 376), M (n = 231) (a), L (n = 303). Tips are colour-coded according to the legend in the left corner; Inner plots indicate the effective population size (i.e. genetic diversity) of OROV infections (Log scale) over time estimated under the coalescent-based Bayesian Skygrid (BSKG) model (posterior median = solid lines, 95% HPD = pale areas) for each segment; (d–f) Regression of sequence sampling dates against root-to-tip genetic distances in a maximum likelihood phylogeny of the Brazilian 2022–2024 expansion clade (n = 254).

Considering the segmented nature of the OROV genome, we investigated potential reassortment and recombination events. We identified 21 reassortment events, including 17 between the S and M segments, seven between the S and L segments, and 11 between the M and L segments. To assess the recent evolution of the three OROV genomic segments, we estimated phylogenetic trees to determine their relationships with other isolates. The novel OROV genome sequences sampled in 2024 clustered with sequences from the 2022–2024 epidemic, forming a monophyletic group with strong bootstrap support (values of 1.0) across all three genomic segments (Fig. 2a-c).

This clade included a basal sequence from Tefé, Amazonas state, sampled in 2015, suggesting a likely Amazonian origin, and has since expanded to other regions, with a north-to-south movement across Brazil (Fig. 2a-c). Analysis of the epidemic expansion, based on estimates of effective population size, revealed a sharp increase at the beginning of 2024 (Fig. 2a-c, inner plots), corresponding to the national surge in OROV cases (Fig. 1).^8^ Phylogenetic trees for the S, M, and L segments (Fig. 2a-c) identified two main lineages: one primarily composed of sequences from the northern and northeastern regions, and another associated with the southeastern and southern regions. The topological discordance among the phylogenetic trees of different genomic segments suggests the occurrence of multiple reassortment events.

**Figure 3:**
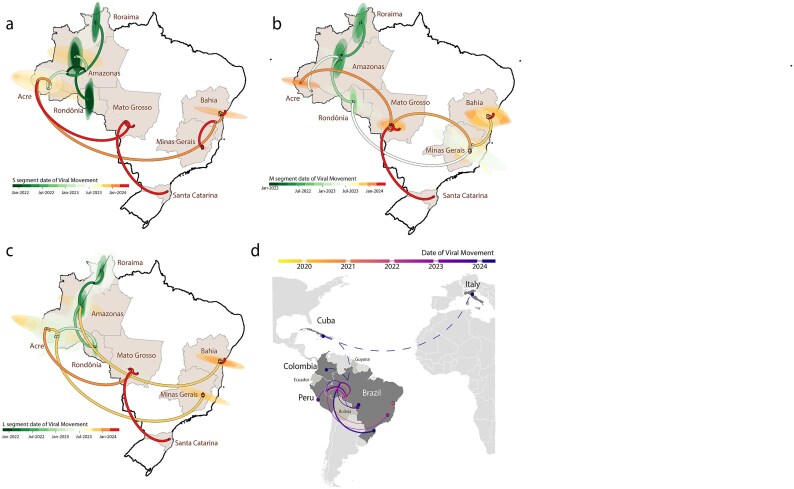
**Inferred Viral Dissemination Patterns of OROV in Brazil and Globally.** (a–c) Phylogeographic reconstruction of the spread of OROV (segments S, M, and L) in Brazil. Circles represent nodes of the maximum clade credibility phylogeny and are coloured according to their inferred time of occurrence. Shaded areas represent the 80% highest posterior density interval, depicting the uncertainty of the phylogeographic estimates for each node. Solid curved lines denote the links between nodes and the directionality of movement; (d) Dissemination patterns of OROV within the Americas and Europe, inferred from ancestral-state reconstructions and annotated by region. Destination countries of viral exchange routes are marked with dots, while curved lines represent movement from the country of origin to the destination in a counterclockwise direction. Dashed lines indicate probable dispersal routes inferred in the absence of genomic evidence, particularly for Cuba and Colombia, based on available travel data. Countries that have reported OROV cases but lack genomic data (Ecuador, Bolivia, and Guyana) are highlighted in light grey to provide epidemiological context.

To reconstruct viral movements across Brazil, we utilized smaller data sets from each genomic segment individually, focusing on the Brazilian 2022–2024 sublineage (Fig. 2a-c). A strong correlation between sampling time and root-to-tip divergence in all three data sets (Fig. 2) allowed for the use of molecular clock models to infer evolutionary parameters. The estimated mean time of origin for the Brazilian 2022–2024 sublineage was early November 2021, with a 95% highest posterior density (HPD) interval from early August 2021 to early January 2022. This sublineage, likely introduced in early 2015, remained undetected due to insufficient active surveillance at the national level. It initially spread from the northern Amazon basin, subsequently reaching the northeastern (Bahia state), midwestern (Mato Grosso state), southeastern (Minas Gerais state), and southern (Santa Catarina state) regions (Fig. 3a-c).

Finally, we analysed the Brazilian 2022–2024 sublineage dataset, incorporating recently isolated international genome sequences from Peru and Italy. Phylogeographic reconstruction revealed a north-to-south viral spread within Brazil, followed by cross-border movement into Peru (Fig. 3d). Additionally, case reports in Italy were linked to returning travellers from Cuba, however, genomic data from Cuba remain unavailable. Travel data from Colombia suggest a possible viral introduction from Brazil, but no genomic sequences from this region are currently available. To account for these limitations, Fig. 3d illustrates probable international dispersal routes inferred in the absence of genomic data, particularly for Cuba and Colombia, using available travel data. Countries reporting OROV cases but lacking genomic sequences—Ecuador, Bolivia, and Guyana—are highlighted in light grey to provide epidemiological context for potential viral circulation in these regions.

## Discussion

The spread of the novel reassortant OROV lineage in the Brazilian Amazon region between 2022 and 2024 underscores its emergence potential and the significant role of ecological and human factors in its dissemination.^11^^,^^12^ Our study, along with recent findings,^11^^,^^12^ highlights the importance of genomic surveillance and phylodynamic analysis in tracing the origin and movement of viral pathogens. Phylogenetic analyses revealed that OROV sequences sampled between 2022 and 2024 formed a highly supported monophyletic clade, tracing their origins to a basal sequence from Tefé, Amazonas, Brazil, sampled in 2015. This supports an Amazonian origin for this clade and aligns with the emergence of a reassortant lineage containing the M segment from viruses in the eastern Amazon region and the L and S segments from viruses in Peru, Colombia, and Ecuador.^10^^,^^11^ This lineage appears to have emerged in central Amazonas between 2010 and 2014, with evidence of long-range silent dispersion during the late 2010s.

Our study identified ~21 reassortment events among different genome segments, highlighting the role of reassortment in OROV's evolutionary history.^11^ These events may have enhanced the virus's adaptability to new ecological niches and hosts, facilitating its spread across diverse regions of Brazil. The sharp increase in OROV cases across multiple regions, along with the concurrent rise in real-time RT-PCR testing, reflects both heightened surveillance and improved detection capabilities. Between 2020 and 2024, the positivity rate nearly doubled, rising from 5.9% to 10%, emphasizing the effectiveness of testing efforts in uncovering previously cryptic transmission. Our genome-based surveillance revealed a clear north-to-south movement of the virus within Brazil, with transmission peaks coinciding with the Amazon basin's rainy season. Environmental factors, including deforestation and climate change, played a significant role in these dynamics.^15^^,^^32^^,^^33^ Recent eco-epidemiological studies emphasize a multi-stage expansion of OROV within Brazil, showing that the virus preferentially circulates in areas of high population density, favourable climatic conditions, and reduced evergreen broadleaf forest cover.^15^ Deforestation, agricultural practices (e.g. banana and cocoa cultivation), and urbanization create optimal mosquito breeding grounds, amplifying OROV transmission risks and shaping its geographical range.^15^ These changes, coupled with rising temperatures and altered weather patterns due to climate change, facilitate vector migration and proliferation into previously unaffected regions.^33–39^ The prolonged cryptic circulation of OROV highlights the need for robust active screening programs to effectively monitor and control its spread.

The detection of three fatal OROV cases, even in patients without comorbidities, suggests a broader clinical impact than previously recognized. Further research into adverse pregnancy outcomes and the potential for vertical transmission is critical. The Brazilian Ministry of Health (BrMoH) recently reported the first fetal death due to OROV with mother-to-child transmission in Pernambuco, underscoring the urgency of understanding factors contributing to severe outcomes and developing effective interventions.^40^^,^^41^

Additionally, our findings indicate that human mobility has played a major role in OROV dispersal beyond Brazil, facilitating its international spread into Peru, Cuba, and Europe. Travel-associated viral dissemination is increasingly recognized as a major factor in arbovirus epidemiology, particularly for emerging and re-emerging pathogens. While our genomic data confirmed OROV’s movement from Brazil to Peru, travel data further suggest viral introduction into Colombia, although no genomic sequences from this region are currently available. Similarly, case reports in Italy were linked to returning travellers from Cuba, but no genomes have been sequenced from Cuba to confirm viral circulation. The absence of genomic data from key regions, including Colombia and Cuba, limits our ability to fully reconstruct transmission dynamics, reinforcing the urgent need for improved genomic surveillance in affected areas. The interplay of climate change, ecological shifts, and human movement highlights the global implications of OROV emergence. The increasing frequency of arbovirus spillover events into previously unaffected areas necessitates coordinated international efforts to monitor and control OROV and other emerging arboviruses. Our findings, combined with recent research, underscore the need for continuous genomic surveillance to unravel the evolutionary and epidemiological patterns of OROV. Such efforts are crucial for anticipating and mitigating future outbreaks, ensuring timely and effective public health responses locally and globally. This comprehensive approach will be instrumental in managing the ongoing spread of OROV and preparing for future threats from similar pathogens.

## Limitation

This study has some limitations that warrant consideration. First, our genomic dataset primarily captures symptomatic cases, as only individuals seeking healthcare and undergoing laboratory testing were included. This likely leads to an underrepresentation of subclinical or mild infections, which may contribute to a broader, undetected transmission of OROV. Furthermore, the paucity of available whole-genome sequences from other locations reporting OROV-positive cases limits the resolution of our phylogeographic analyses and hinders the ability to finely reconstruct the directions and patterns of virus dispersion. A more extensive dataset from multiple affected countries would be essential to differentiate between travel-related introductions and sustained transmission within novel locations. Such data would also improve our ability to assess how OROV spreads internationally and whether specific lineages are more likely to establish endemicity in new regions. Lastly, while this study identifies point mutations in OROV genomes, particularly those associated with fatal cases, further functional studies are necessary to determine whether these mutations influence viral virulence, replication capacity, or other phenotypic traits. Addressing these gaps in future research will be critical for a more comprehensive understanding of OROV transmission dynamics, pathogenicity, and its potential for international spread.

## Supplementary Material

Figure_S1_taaf018

Figure_S2_taaf018

Figure_S3_taaf018

Table_S1_taaf018

Supplementary_figure_legends_taaf018

## Data Availability

All sequences generated in this study, along with their GenBank accession numbers and associated metadata, are provided in Table S1. For segment S accession number (Additionally, all input files and scripts used for the analyses are publicly available on GitHub at https://github.com/genomicsurveillance/OROV.
