# Supplementary figures and images for: Travel-associated international spread of Oropouche virus beyond the Amazon
Source: J Travel Med. 2025 Mar 2;32(3):taaf018. doi: 10.1093/jtm/taaf018 (PMC11955161; doi:10.1093/jtm/taaf018)

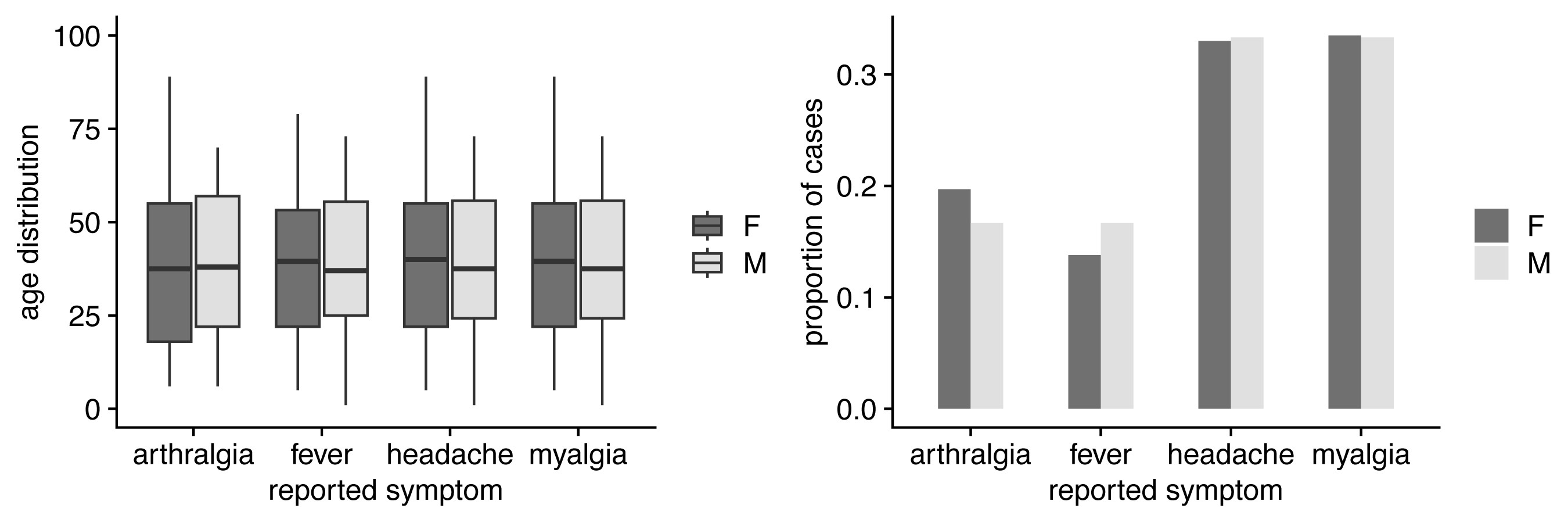

Supplement: Figure_S1_taaf018 [file figure_s1_taaf018.jpeg]

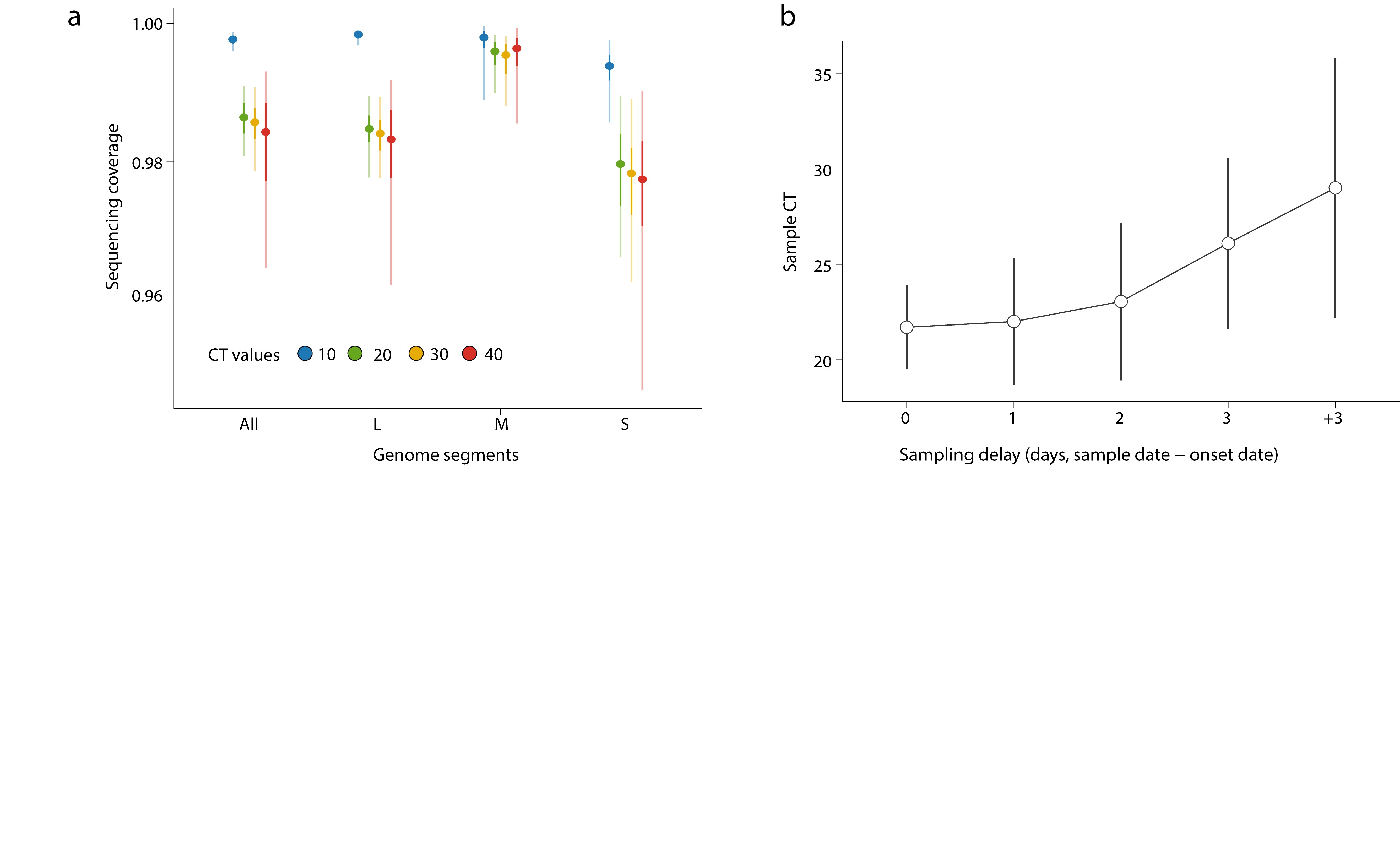

Supplement: Figure_S2_taaf018 [file figure_s2_taaf018.jpeg]

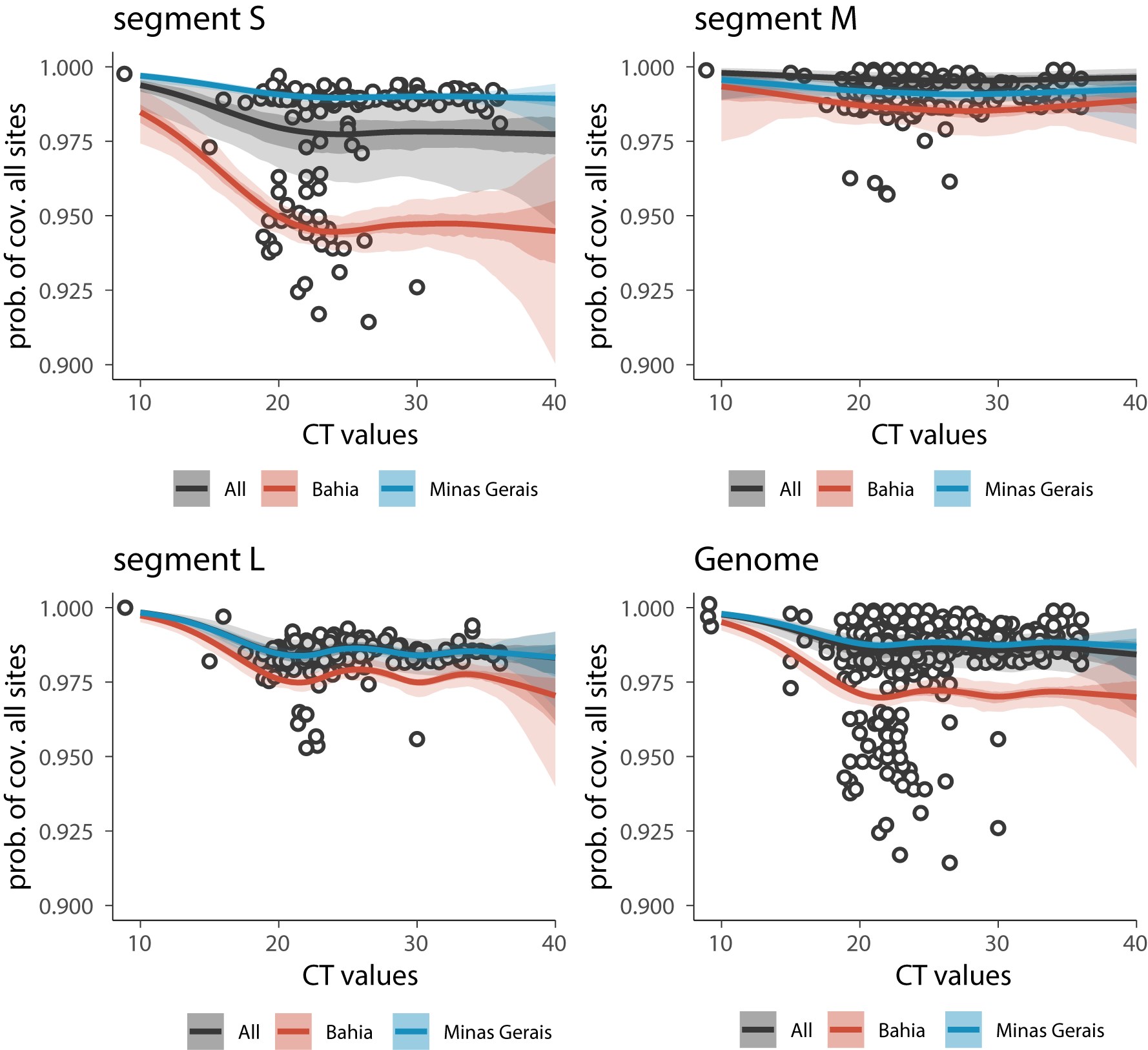

Supplement: Figure_S3_taaf018 [file figure_s3_taaf018.jpeg]
