# Supplementary material for: Travel-associated international spread of Oropouche virus beyond the Amazon
Source: J Travel Med. 2025 Mar 2;32(3):taaf018. doi: 10.1093/jtm/taaf018 (PMC11955161; doi:10.1093/jtm/taaf018)
Supplement: Supplementary_figure_legends_taaf018 [file supplementary_figure_legends_taaf018.docx]

**Figure S1. Symptoms versus age and gender.** The (left) age distribution of cases reported with different symptoms and (b) proportion of cases reporting different symptoms, both disaggregated by gender. Data includes the metadata for the sequenced samples.

**Figure S2. Sequencing Coverage and Sample CT Value Analysis: Model Estimates.** a) Summary of estimated sequence coverage dependent on sample CT using a Generalized Additive Model. Shown are the mean (points) and 95% CI (ranges) for the model estimates given selected values of CT (10, 20, 30, 40, in color) for all segments separately and together; b) Sample CT values (mean as points, standard deviation as ranges) plotted against the sampling delay (days between symptom onset and sample collection).

**Figure S3. Sequence Coverage Probability Across Genome Segments in Different Brazilian States.** The figure displays the probability of coverage for all sites across four different genome segments (S, M, L, and the entire genome) in relation to the CT value. The probability was estimated using a Generalized Additive Model (GAM; as done previously e.g. for CHIKV sequencing data [Giovanetti et al Emerg Infect Dis. 2023;29(9):1859-1863]). The GAM was defined with a binomial family, with coverage modeled as the proportion of sites recovered from sequencing, using sample state (Brazilian state) as random effect. Solutions presented (in color) include the specific ones for the two states with most sequencing (Bahia, red; Minas Gerais, blue) and the general model output (gray).
